# *CCAT2*, a novel long non-coding RNA in breast cancer: expression study and clinical correlations

**DOI:** 10.18632/oncotarget.1292

**Published:** 2013-09-18

**Authors:** Roxana S Redis, Anieta M Sieuwerts, Maxime P Look, Oana Tudoran, Cristina Ivan, Riccardo Spizzo, Xinna Zhang, Vanja de Weerd, Masayoshi Shimizu, Hui Ling, Rares Buiga, Victor Pop, Alexandru Irimie, Riccardo Fodde, Isabella Bedrosian, John WM Martens, John A Foekens, Ioana Berindan-Neagoe, George A Calin

**Affiliations:** ^1^ Department of Experimental Therapeutics, The University of Texas MD Anderson Cancer Center, Houston, Texas, USA; ^2^ Department of Medical Oncology, Erasmus MC Cancer Institute, Erasmus University Medical, Rotterdam, The Netherlands; ^3^ Department of Molecular Science, University of Medicine and Pharmacy Iuliu Hatieganu, Cluj-Napoca, Romania; ^4^ Department of Functional Genomics, The Oncology Institute, Cluj-Napoca, Romania; ^5^ Division of Experimental Oncology, CRO, National Cancer Institute, Aviano, Italy; ^6^ Center for RNA Interference and Non-coding RNAs The University of Texas MD Anderson Cancer Center, Houston, Texas, USA; ^7^ Department of Pathology, Josephine Nefkens Institute, Erasmus Medical Center, Rotterdam, The Netherlands; ^8^ Department of Surgical Oncology, The University of Texas MD Anderson Cancer Center, Houston, Texas, USA; ^9^ Research Center for Functional Genomics, Biomedicine and Translational Medicine, University of Medicine and Pharmacy Iuliu Hatieganu, Cluj-Napoca, Romania; ^10^ Department of Pathology, The Oncology Institute, Cluj-Napoca, Romania; ^11^ Department of Surgical and Gynecology Oncology, University of Medicine and Pharmacy Iuliu Hatieganu, Cluj-Napoca, Romania

## Abstract

The clinical outcome of BC patients receiving the same treatment is known to vary considerably and thus, there is a compelling need to identify novel biomarkers that can select the patients that would benefit most from a given therapy and can predict the clinical outcome. The aim of this study was to determine the prognostic value of *CCAT2*, a novel long ncRNA recently characterized by our group and overlapping SNP rs6983267, in BC patients. We first evaluated by RT-qPCR and ISH the expression of *CCAT2* in normal breast tissue and BC tissue and further analyzed *CCAT2* expression in an independent set of 997 primary BC with regard to clinical, histological, pathological and other biological factors. Also, we explored the possibility of *CCAT2* adding to the prognostic value of multivariate models that already included the traditional prognostic factors. Finally, we identified in *in vitro* models the impact of *CCAT2* expression and SNP rs6983267 genotype on cell migration and chemoresistance. Our results revealed that although overexpressed in BCs in two out of three sets of patients, and having the highest expression in lymph node negative (LNN) disease, *CCAT2* expression levels are informative solely for a subgroup of BC patients, namely for patients with LNP disease that have received adjuvant CMF chemotherapy. For this subgroup high levels of *CCAT2* suggest the patients will not benefit from CMF containing adjuvant chemotherapy (shorter MFS and OS). Additionally, we found that *CCAT2* upregulates cell migration and downregulates chemosensitivity to 5'FU in a rs6983267-independent manner.

## INTRODUCTION

Evidence is rapidly accumulating that, in addition to short microRNAs, long non-coding RNAs (lncRNAs, transcripts of at least 200 nt long that do not code for proteins but regulate expression of coding genes) are involved in human tumorigenesis. Their ability to regulate essential pathways for tumor initiation and progression together with their tissue and stage specificity, promotes them as valuable biomarkers and therapeutic targets [1-5]. In an earlier study our group demonstrated that a large fraction of genomic ultraconserved regions (UCRs) encode a particular set of ncRNAs, named transcribed UCRs (T-UCRs) whose expression is altered in human cancers [6]. Genome-wide profiling revealed that T-UCRs have distinct signatures in human leukemias and carcinomas and they are frequently located at fragile sites and genomic regions involved in cancers. Our findings argued that ncRNAs are involved in tumorigenesis to a greater extent than previously thought. This offers the prospect of defining tumor-specific signatures of ncRNAs that are associated with diagnosis, prognosis, and response to treatment.

Chromosomal copy number aberrations (CNAs) are common in breast cancer (BC) and involve genomic regions in a frequency and combination that suggest distinct routes of tumor development. Patterns of copy number gains and losses define breast tumors with distinct clinico-pathological features and patient prognosis [7, 8]. For example, the 5-year survival rates varied from 96% in a group of BCs defined by +1q, +16p, and -16q to 56% in a group of BCs defined by -8p and +8q. These correlations were independent of nodal status, tumor size, and progesterone receptor (PR) status in a multivariate analysis [9]. Furthermore, amplification of 8q24 genomic region was observed more frequently in invasive solid-tubular or scirrhous tumors (48/92, 52%) than in less aggressive histological types (7/25, 28%) [10]. In another study results suggested that there was a relationship between 8q24 DNA amplification profiles and breast tumor phenotype [11]. Thus, amplification of oncogene(s) located on 8q24 may play a role in the development and/or progression of a substantial proportion of primary breast cancers, particularly those of the invasive histology, but the nature of this/these genes is yet unknown.

We have recently reported the discovery of a novel long ncRNA, *CCAT2* (Colon Cancer Associated Transcript 2) transcribed from 8q24 genomic region [12]. The *CCAT2* genomic locus similar to UCRs is highly conserved and harbors the SNP rs6983267, which was shown to be associated with predisposition to colon, ovarian and prostate cancer [13-18] and more recently with risk of metastasis in inflammatory BC [19]. *CCAT2* promotes metastasis and chromosomal instability in microsatellite stable (MSS) colon cancer through a mechanism involving transcription factors, oncogenes and microRNAs [12]. In light of these findings and previous reports, we hypothesized that *CCAT2* may be overexpressed in BC and act as an oncogene inducing a metastatic phenotype. To investigate this hypothesis, we evaluated the expression of *CCAT2* in non-cancer and BC tissues and, in a large independent set of primary tumors the related expression with clinical, histological, pathological and other biological factors. Moreover, we tested expression levels of *CCAT2* in multivariate models that already included the traditional prognostic factors. Finally, we expanded our study to include *in vitro* models, in which we evaluated the impact of *CCAT2* expression and the SNP rs6983267 on cell migration and chemoresistance.

## RESULTS

### CCAT2 is expressed in breast tumors

**Figure 1:**
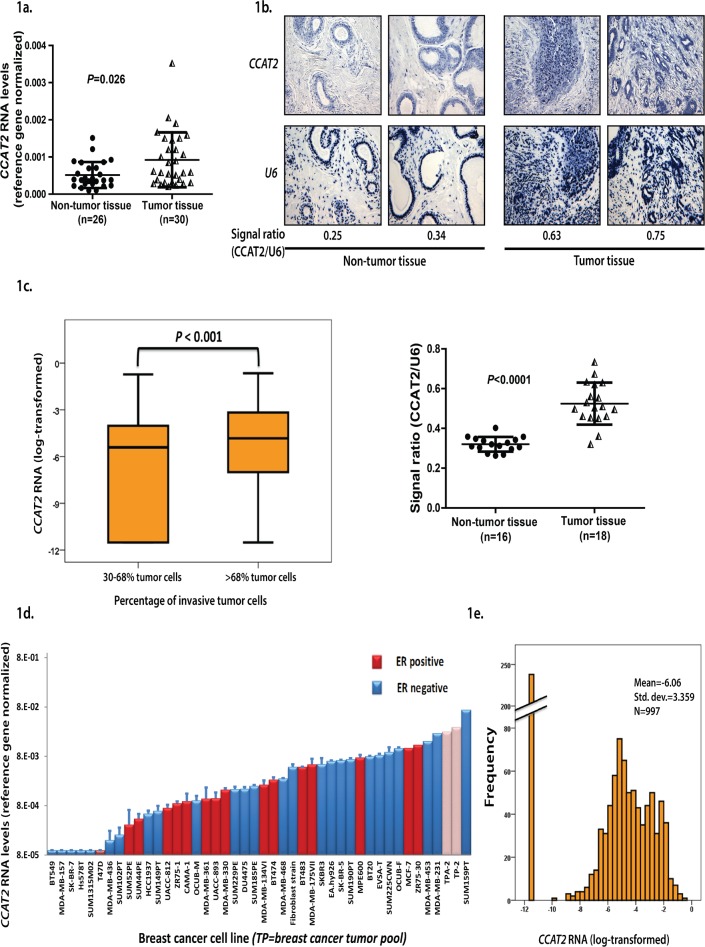
a) CCAT2 expression in BC patients (OICN cohort) quantified by qRT-PCR. b). *In situ* hybridization of *CCAT2* and *U6* (as reference) in BC patient samples and *CCAT2* expression in BC patients determined by quantifying ISH images from TMA slide. Each sample was represented in 5 replicates for non-cancer tissue and 3 replicates for cancer tissue on the slide. c). Expression of *CCAT2* mRNA in clinical breast cancers (EMC cohort) after dividing the cancers at the median level in tumors containing a relatively high percentage (>68%) of invasive tumor cells and tumors containing a relatively low percentage (30-68%) of invasive tumor cells (RT-qPCR mRNA levels expressed as fold difference relative to the 3-gene reference gene set). d). Expression levels of *CCAT2* mRNA in human breast cancer cell lines and 2 pools of breast cancers (TP-2 and TPA-2, hatched bars) (RT-qPCR mRNA levels expressed as fold difference relative to the 3-gene reference gene set). e) Distribution of *CCAT2* mRNA in clinical breast cancers (RT-qPCR levels expressed relative to the 3-gene reference gene set).

While focusing on the genomic characterization of *CCAT2* novel long non-coding RNA, the Northern Blot data showed that it is expressed also in BC cell lines (Supplementary Fig. 1). We further measured the RNA expression levels of *CCAT2* by RT-qPCR in a set of 56 unmatched samples (26 non-cancer breast tissues and 30 breast cancer tissues) from OICN and detected significantly increased levels of *CCAT2* RNA in tumor samples compared to the non-tumor group (*P*=0.026) (Fig. 1a).

To further explore this result and identify the tissue localization of *CCAT2*, we performed *in situ* hybridization (ISH) employing a locked-nucleic acid (LNA) specific probe for the lncRNA on tissue microarray (TMA), containing 16 non-tumor samples and 18 tumor samples from MDACC. Notably, these patients were not subjected to neo-adjuvant chemotherapy. All the samples, both tumor and non-tumor tissues, showed a strong staining of *CCAT2* in epithelial cells and a less intense staining in inflammatory and stromal cells. *CCAT2* appeared to have higher expression in the epithelial component of BC tissue than in epithelial component of non-tumor tissue (*P*<0.001) (Fig. 1b). Moreover, expression was detected in invasive epithelial components, as well as in “*in situ*” epithelial lesions, with no visible differences between the two components of cancers (Supplementary Fig. 2a, b). Apocrine metaplasia, columnar metaplasia and the usual intraductal epithelial hyperplasia disclosed a similar pattern of *CCAT2* expression as the non-tumor breast tissue (Supplementary Fig. 2c). The ISH assay allowed also for the assessment of the subcellular localization of *CCAT2*, expression was detected in both the nucleus and the cytoplasm, with a more intense staining of the nucleus, indicating an obvious enrichment of *CCAT2* in the nuclear compartment (Supplementary Fig. 2d).

However, in a different set of 15 unpaired normal breast tissues from EMC, *CCAT2* expression levels measured by RT-qPCR did not vary significantly from the levels measured in the n=977 clinical specimens (*CCAT2* RNA levels in normal and tumor tissue, average ± SD: 0.0078±0.00445 and 0.0060±0.00298, respectively, *P*>0.05). Although, after dividing the tumors at the median in groups containing a low (n=492, 30-68%) or high (n=505, >68%) percentage of invasive tumor cells, *CCAT2* RNA levels were significantly higher in the group of tumors with high invasive tumor cells (Mann-Whitney U Test, *P*<0.001) (Fig 1c). Therefore, additional larger studies are needed to assess the levels of *CCAT2* in breast tumors versus normal tissues in multiple patient populations.

Additionally, we assessed *CCAT2* expression in a set of cultured breast cell lines, showing a wide range of expression levels with the expression measured in 2 different pools of BCs tissues located in the upper range (hatched bars, Fig. 1d). Correspondingly to our observation in cultured cell lines (Fig. 1d, 6 out of 40, 15%), levels of *CCAT2* were undetectable within 35 amplification rounds in 238 out of 997 (24%) of the primary breast tumors from the EMC patient set (Fig. 1e). This patient set of *CCAT2* expressers was further used for investigating the correlations between *CCAT2* and clinical, histo-morphological and biological characteristics.

### Associations of CCAT2 with relevant biological factors, amplification of 8q24 and the SNPs rs6983267 and rs13281615

**Figure 2:**
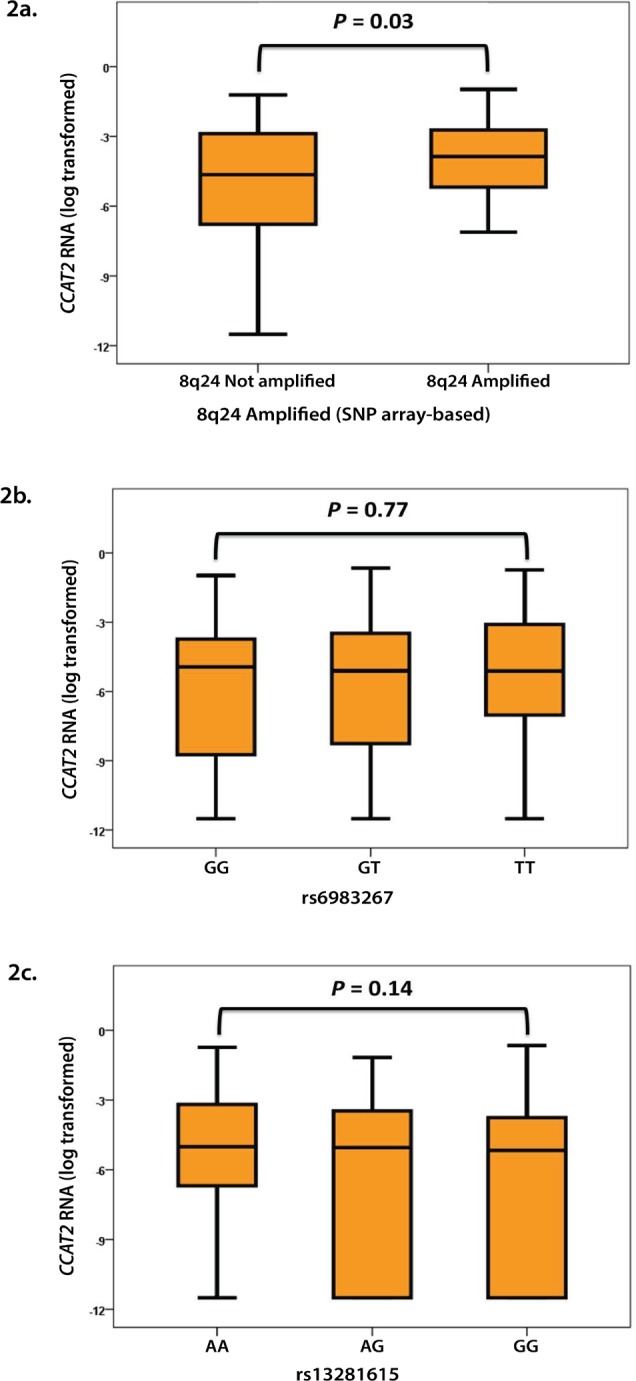
Associations of *CCAT2* with a) amplification of 8q24 (n=80 amplified versus n=146 unamplified) and the SNPs b) rs6983267 (n=241 GG, n=393 GT, n=238 TT) and c) rs13281615 (n=297 AA, n=392 AG, n=157 GG).

To investigate whether there is an association of *CCAT2* expression levels with well-established biological factors, we matched our *CCAT2* expression data with those of *ESR1*, *PGR*, *ERBB2*, and the proliferation marker *Ki-67* measured in the same preparations (EMC patient set). In addition, we used our SNP data to associate *CCAT2* transcript levels in 226 LNN patients with known DNA copy number to identify tumor samples with copy number alterations that showed concordant *CCAT2* gene expression alterations. In these clinical samples, increasing levels of *ESR1* and *PGR* associated significantly with decreasing levels of *CCAT2* (Spearman *r*_*s*_ = -0.14 and -0.13, respectively, n=997, *P*<0.001), although *CCAT2* was not significantly (*P*=0.79) associated with *ERBB2* (Mann-Whitney U test for amplified vs. unamplified *ERBB2*) or *Ki-67* (Spearman *r*_s_ = 0.022, n=988, *P*=0.50). As expected due to the genomic location, a positive association with amplification of the 8q24 region was observed (*P*=0.03 in Mann-Whitney U test with 80 out of 226 samples amplified in the 8q24 region covering the *CCAT2* gene) (Fig. 2a). Increasing levels of *MYC*, also located on 8q24, were positively associated with *CCAT2* (Spearman *r*_*s*_ = 0.11, n=992, *P*<0.001).

Next, we investigated whether there is an association between the expression levels of *CCAT2* and SNP rs6983267. We measured *CCAT2* expression and genotyped 872 of our primary breast tumor samples, but did not find a significant correlation (Fig. 2b). It must be however mentioned that in this clinical BC cohort there was a significant deviation from the Hardy-Weinberg equilibrium (HWE *P*=0.004) possibly implying a selection bias. Of note that in this cohort there was also no significant correlation for *CCAT2* and the different genotypes of SNP rs13281615. This SNP, for which our measurements did not result in a deviation from the Hardy-Weinberg equilibrium (HWE *P*=0.17), is also located in the 8q24 region, approximately 10 kb upstream of *CCAT2* and the SNP rs6983267, and has previously been associated with increased BC risk [13, 20] (Fig. 2c).

### Associations of CCAT2 RNA with clinical and histo-morphological factors and risk to develop a distant metastasis

Correlations of RNA expression of *CCAT2* with patient and tumor characteristics are shown in Table 1. Most notable were the strong inverse associations of *CCAT2* expression levels with nodal status (*P*<0.001) and hormone (ER and PR) receptor status (*P*<0.001).

**Table 1:** Associations of *CCAT2* expression levels with clinical, biological and histo-morphological factors.

	All patients			
Characteristic	No of patients 997	*%* *100%*	***CCAT2* RNA** (×10^−2^)	
All patients in this cohort	997	*100%*	median 0.61	inter-quartile 2.98
**Age (years)**				
≤40	140	*14%*	0.69	5.12
41-55	394	*40%*	0.57	3.07
56-70	313	*31%*	0.56	2.36
>70	150	*15%*	0.77	2.82
			*P* =0.19^†^	
**Menopausal status**				
premenopausal	460	*46%*	0.61	3.69
postmenopausal	537	*54%*	0.59	2.40
			*P* =0.06^§^	
**ER mRNA status^≠^**				
negative, < 0.2	230	*23%*	1.19	4.78
positive, ≥0.2	767	*77%*	0.54	2.16
			***P* <0.001^‡^**	
**PR mRNA status^≠^**				
negative, < 0.1	396	*40%*	0.83	4.09
positive, ≥ 0.1	601	*60%*	0.49	2.24
			***P* <0.001^‡^**	
**Grade**				
Poor	531	*53%*	0.65	3.44
Unknown	282	*28%*	0.56	2.41
Moderate/good	184	*18%*	0.57	2.44
			*P* =0.38^║^	
**Tumor size**				
pT1, ≤2 cm	377	*38%*	0.57	2.20
pT2, >2-5 cm + unknown	534	*54%*	0.64	3.46
pT3, >5 cm + pT4	86	*9%*	0.52	2.59
			*P* =0.24^║^	
**Lymph nodes involved**				
no, (LNN)	621	*62%*	0.84	4.60
yes, (LNP), 1 to 3	189	*19%*	0.40	1.24
yes, (LNP), >3	187	*19%*	0.45	1.11
			***P* <0.001^║^**	
**Histological type^†^**				
IDC	533	*53%*	0.56	2.39
DCIS + IDC	151	*15%*	0.50	1.97
ILC	85	*9%*	0.61	3.58
mucinous	29	*3%*	0.47	1.27
			*P* =0.50^║^	

≠ ER+ and/or PR+ with RT-PCR cut point used for ER, 0.2 and PR, 0.1 (relative to reference gene set).

† Only data for the 4 most common histological subtypes are presented in this table, IDC; infiltrating ductal carcinoma, DCIS; ductal carcinoma in situ, ILC; infiltrating lobular carcinoma.

* Due to missing data numbers do not add up to 997.

‡ *P* for Spearman rank correlation test.

§ *P* for Mann-Whitney U test.

║ *P* for Kruskal-Wallis test, including a Wilcoxon-type test for trend when appropriate.

Within our evaluation of the possible relationship of *CCAT2* levels with prognosis we proceeded with performing exploratory Cox univariate analyses for metastases-free survival (MFS) and overall survival (OS) as a function of *CCAT2* RNA levels (divided in 4 equally sized groups based on the quartile levels of all 997 tumors) in the clinically relevant subgroups of LNN, LNP, ER+, and ER- patients, groups in which we already observed a divergent *CCAT2* expression (Table 2). Moreover, because all patients with LNP disease received systemic adjuvant therapy, we divided these patients according to the adjuvant therapy they had received. Results are summarized in Table 2. Despite the higher expression of *CCAT2* in tumors of the LNN vs. the LNP patient cohort, there was no significant association with prognosis in these patients who had not received systemic adjuvant therapy, neither in the ER-negative subgroup, nor in the ER-positive subgroup. Although *CCAT2* levels were overall not informative for LNP patients that had received adjuvant tamoxifen or an anthracycline-containing regimen, for those LNP patients that had specifically received adjuvant CMF (cyclophosphamide, methotrexate, and 5-fluorouracil (5'FU)), increasing levels of *CCAT2* were associated with shortened MFS (*P*=0.02) and OS (*P*=0.004). Associations of *CCAT2* levels with patient and tumor characteristics for these LNP patients (n=134) that received adjuvant CMF are shown in Supplementary Table 2.

**Table 2:** *CCAT2* expression levels in Cox univariate analysis for distant metastasis-free and overall survival.

***CCAT2* RNA** (divided in 4 equal parts)		**MFS**			**OS**		
Histo-morphological and clinical subgroups	No patients	HR	(95% CI)	*P*	HR	95% CI	*P*
**All patients**	250	1		*0.69*	1		*0.67*
	250	1.13	(0.88-1.47)	*0.34*	1.12	(0.88-1.47)	*0.40*
	248	1.16	(0.89-1.50)	*0.27*	1.13	(0.86-1.48)	*0.37*
	249	1.09	(0.84-1.41)	*0.54*	1.17	(0.90-1.53)	*0.24*
**Nodal status and adjuvant therapy**							
**LNN, no adjuvant**	130	1		*0.50*	1		*0.53*
	138	1.31	(0.89-1.93)	*018*	1.17	(0.79-1.74)	*0.44*
	150	1.27	(0.86-1.86)	*0.23*	1.25	(0.85-1.84)	*0.26*
	203	1.26	(0.88-1.81)	*0.22*	1.29	(0.90-1.86)	*0.16*
**LNP, adjuvant tamoxifen**	59	1		*0.21*	1		*0.07*
	45	0.99	(0.60-1.66)	*0.98*	1.03	(0.61-1.73)	*0.92*
	48	1.17	(0.72-1.90)	*0.54*	1.08	(0.64-1.80)	*0.78*
	15	2.12	(0.06-4.22)	***0.03***	2.62	(0.34-5.13)	***0.01***
**LNP, adjuvant anthracycline**	19	1		*0.13*	1		***0.02***
	24	1.36	(0.65-2.83)	*0.41*	1.82	(0.82-4.03)	*0.11*
	21	0.55	(0.23-1.31)	*0.18*	0.50	(0.18-1.38)	*0.28*
	9	0.68	(0.22-2.11)	*0.50*	**0.45**	(0.10-2.07)	***0.16***
**LNP, adjuvant CMF**	41	1		***0.02***	1		***0.00***
	43	0.94	(0.49-1.80)	*0.85*	1.08	(0.53-2.18)	*0.84*
	29	1.85	(0.97-3.53)	*0.06*	2.34	(1.16-4.70)	***0.02***
	21	2.44	(1.23-4.86)	***0.01***	2.94	(1.43-6.03)	***0.00***
**Estrogen receptor status^a^**							
**ER mRNA negative**	42	1		*0.10*	1		*0.74*
	52	1.57	(0.88-2.81)	*0.13*	1.10	(0.63-1.92)	*0.73*
	56	1.41	(0.79-2.50)	*0.24*	0.95	(0.55-1.66)	*0.86*
	80	0.91	(0.51-1.62)	*0.75*	0.84	(0.50-1.42)	*0.52*
**ER mRNA positive**	208	1		*0.79*	1		*0.60*
	198	1.04	(0.77-1.39)	*0.81*	1.11	(0.82-1.51)	*0.49*
	192	1.08	(0.81-1.45)	*0.59*	1.15	(0.85-1.57)	*0.37*
	169	1.16	(0.86-1.57)	*0.33*	1.24	(0.91-1.70)	*0.18*
**LNN, estrogen receptor status^a^**							
**ER mRNA negative**	22	1		*0.12*	1		*0.55*
	31	1.26	(0.58-2.76)	*0.56*	0.93	(0.43-2.00)	*0.85*
	37	1.07	(0.50-2.30)	*0.86*	0.77	(0.36-1.65)	*0.50*
	66	0.62	(0.29-1.31)	*0.21*	0.64	(0.32-1.30)	*0.22*
**ER mRNA positive**	108	1		*0.18*	1		*0.20*
	107	1.28	(0.82-2.01)	*0.28*	1.20	(0.76-1.91)	*0.43*
	113	1.27	(0.81-1.99)	*0.29*	1.38	(0.88-2.16)	*0.16*
	137	1.59	(1.05-2.41)	***0.03***	1.55	(1.02-2.38)	***0.04***
**LNP, estrogen receptor status^a^**							
**ER mRNA negative**	20	1		*0.09*	1		*0.15*
	21	2.07	(0.87-4.95)	*0.10*	1.43	(0.64-3.19)	*0.39*
	19	2.23	(0.93-5.33)	*0.07*	1.52	(0.68-3.40)	*0.31*
	14	2.98	(1.21-7.35)	***0.02***	2.67	(1.17-6.09)	***0.02***
**ER mRNA positive**	100	1		*0.65*	1		*0.64*
	91	0.88	(0.60-1.30)	*0.53*	1.05	(0.70-1.58)	*0.81*
	79	1.03	(0.70-1.52)	*0.89*	1.05	(0.68-1.62)	*0.82*
	32	1.24	(0.74-2.09)	*0.41*	1.44	(0.84-2.49)	*0.19*

a ER status according RT-qPCR cut point at 0.2 (relative to reference geneset).

### Univariate and multivariate analysis for MFS

To further investigate the independent relationship of *CCAT2* with prognosis for the LNP patients that had received adjuvant CMF, we have extended our previous patient group [12] with 13 new patients, which included 5 new LNP primary BC patients which received systemic adjuvant CMF, and reanalyzed the data based on the most updated clinical information available for these patients. We therefore have redone the Cox univariate analyses for MFS as a function of *CCAT2* expression levels in these LNP primary BC patients which received systemic adjuvant CMF. To visualize the prognostic value of *CCAT2* in Kaplan-Meier curves, we divided *CCAT2* expression into 4 parts (negative, low, intermediate, and high, as based on the quartile levels of all 997 tumors) (Supplementary Fig. 3) for the 134 LNP primary BC patients that received systemic adjuvant CMF. In these updated analyses, the upper 25% (high levels) of *CCAT2* were significantly associated with MFS (HR 2.44, *P*=0.011) (Table 2 and Table 3).

**Table 3:** Cox univariate and multivariate analysis for MFS as a function of *CCAT2* in primary breast tumors from 134 LNP breast tumor patients that received adjuvant CMF^a^

		Univariate analysis			Multivariate analysis		
Factor	No. Patients	HR	95% CI	*P*	HR	95% CI	*P*
					**Base model**		
**Age at start of therapy, years**							
≤40	35	1			1		
>40	99	0.74	(0.44-1.25)	*0.260*	0.73	(0.43-1.26)	*0.259*
**Menopausal status at start of therapy**							
premenopausal	119	1			1		
postmenopausal	15	1.15	(0.57-2.32)	*0.700*	1.40	(0.63-3.08)	*0.410*
**Tumor size**							
pT1, ≤2 cm	36	1			1		
pT2, >2- ≤5 cm	80	1.92	(1.03-3.56)	***0.039***	1.73	(0.92-3.23)	*0.087*
pT3, >5 cm, + pT4	18	2.82	(1.28-6.20)	***0.010***	3.42	(1.49-7.87)	***0.004***
**Lymph nodes involved**							
1−3	92	1			1		
>3	42	1.62	(0.99-2.62)	*0.052*	1.58	(0.96-2.59)	*0.069*
**Grade**							
poor	70	1			1		
unknown	42	0.60	(0.34-1.04)	*0.066*	0.48	(0.26-0.87)	***0.016***
moderate	22	0.46	(0.22-0.96)	***0.039***	0.46	(0.21-0.98)	***0.044***
**ER status, mRNA level^c^**							
negative, <0.2	25	1			1		
positive, ≥0.2	109	0.77	(0.42-1.40)	*0.390*	0.63	(0.28-1.42)	*0.265*
**PR status, mRNA level^c^**							
negative, <0.1	45	1			1		
positive, ≥0.1	89	0.97	(0.59-1.60)	*0.910*	1.48	(0.73-2.99)	*0.273*
					**Additions to the base model^b^**		
***CCAT2* RNA level**							
0-25%	41	1			1		
25-50%	43	0.94	(0.49-1.80)	*0.846*	0.98	(0.50-1.93)	*0.960*
50-75%	29	1.85	(0.97-3.53)	*0.063*	1.94	(0.98-3.85)	*0.056*
75-100%	21	2.44	(1.23-4.86)	***0.011***	2.25	(1.07-4.74)	***0.033***

a Seven of these patients received both hormonal therapy and chemotherapy and one patient had a ovarectomy.

b CCAT2 RNA levels were separately introduced to the base multivariate model that included the following factors: age, menopausal status, nodal status, pathological tumor size, grade, ER and PR status.

c ER and PR status according RT-qPCR cut point at 0.2 for ER and 0.1 for PR (mRNA levels relative to reference gene set).

Next, *CCAT2* was also separately introduced to the base multivariate model that included the factors age, menopausal status, nodal status, tumor size, grade, ER and PR. Expression levels of *CCAT2* also contributed significantly to the multivariate model for MFS in these LNP patients that had received adjuvant CMF (HR 2.25, *P*=0.033 for the upper vs. the lowest group) (Table 3).

### CCAT2 RNA, but not the SNP rs6983267 modulates cell migration and chemosensitivity *in vitro*

**Figure 3:**
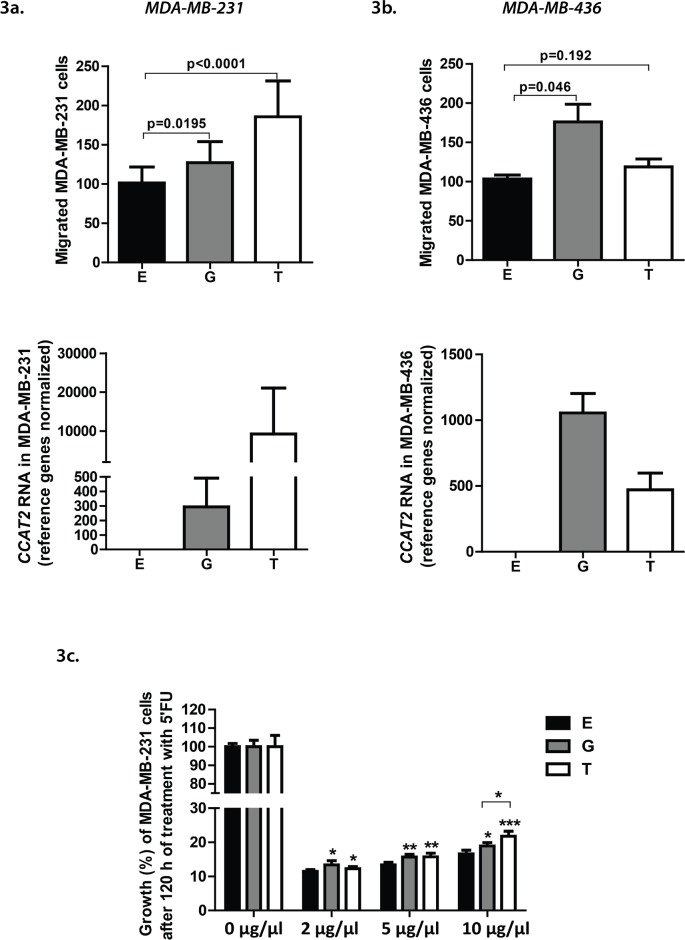
a). (Upper panel) Migration of MDA-MB-231 cells stably transfected with the empty vector (E), and vectors containing the *CCAT2* G, T alleles. Results represent the mean value of 3 experiments performed in triplicate ± SD. (Lower panel) *CCAT2* expression levels in the MDA-MB-231 clones assessed by RT-qPCR (*U6* and *HPRT1* were used as reference genes). b). (Upper panel) Migration of MDA-MB-436 cells transiently transfected with pcDNA *CCAT2* vectors. Results represent the mean value of 3 experiments performed in triplicate ± SD. (Lower panel) *CCAT2* expression levels in the MDA-MB-436 cells assessed 24 hours after transient transfection with pcDNA *CCAT2* vectors by qRT-PCR qPCR (*U6* and *HPRT1* were used as reference genes). c). Effect of 5'Fluorouracil (5'FU) on cell proliferation of MDA-MB-231 *CCAT2* clones (E, G, T). Cells were treated with 3 different concentrations of 5'FU and after 120 hours and the cell viability was determined by the MTT assay. Results represent the mean value of 2 experiments performed in quadruplicate ± SD. Statistical significance is marked with the star symbol, namely ‘*’ for *P*<0.05, ‘**’ for *P*<0.001 and ‘***’ for *P*<0.0001.

We next aimed to explore *in vitro* the biology behind the results we obtained from the EMC patients cohort and for this purpose we cloned *CCAT2* in a retroviral expression vector and transfected MDA-MB-231, a basal-like BC cell line (endogenous rs6983267 TT genotype). We generated *CCAT2* overexpressing clones that distinctively overexpress the two alleles (G and T) of the SNP rs6983267, for assessing their individual impact onto cell migration and chemoresistance. High levels of the *CCAT2* transcript induced a higher migratory potential of the cells independent of the genotype. We observed a 30% increase in migration for *CCAT2* G-overexpressing cells (*P*=0.0195), while *CCAT2* T-overexpressing cells revealed a 70% increase (*P*<0.0001) compared to the control cells (Fig. 3a, upper panel), simultaneously suggesting a dose-dependent effect (Fig. 3a, lower panel). To further confirm the result, we transiently transfected MDA-MB-436 cells, also an ER-negative basal-like BC cell line (endogenous rs6983267 TT genotype), but with much lower levels of endogenous *CCAT2* (Fig. 1c), with the G and T *CCAT2* pcDNA 3.1 vectors and performed migration assays. While *CCAT2* overexpressing MDA-MB-436 G cells migrated significantly more (*P*=0.046) compared to control cells, the migration of MDA-MB-436 T cells increased compared to control cells, but not statistically significant (*P*=0.192). However, the migration and RT-qPCR results taken together suggest that the G allele could induce a stronger migratory phenotype in this cell line than the T allele (Fig. 3b).

Considering that the clinical data analysis disclosed a significant correlation between the expression of *CCAT2* and the MFS and OS of patients who received adjuvant CMF chemotherapy, we sought to determine whether these results are reproducible *in vitro* and whether the two alleles would modulate differently the chemosensitivity of the cells. Therefore, 3-[4,5-dimethylthiazol-2-yl]-2,5-diphenyltetrazolium bromide (MTT) assays were performed to determine the viability of MDA-MB-231 *CCAT2* clones treated with the DNA-damaging agent 5'FU, one of the components of CMF. Three different drug concentrations were administrated and 120 hours after treatment, the chemosensitivity was assessed. For all the drug concentrations, both the G and the T *CCAT2* clones displayed significantly lower chemosensitivity compared to the control cells (Fig. 3c).

## DISCUSSION

The objective of this study was to determine the prognostic value of *CCAT2*, a long non-coding RNA recently characterized by our group and located in a highly conserved genomic region [12]. Although, *CCAT2* is overall overexpressed in BCs in two out of three patients sets, having the highest expression in LNN disease, its expression levels are clinically informative solely for a subgroup of BC patients, namely for patients with LNP disease that have received adjuvant CMF chemotherapy. This indicates that *CCAT2* is not a pure prognostic marker for BC progression, but for a particular subgroup (LNP, CMF treated patients) the expression level of *CCAT2* may predict metastasis and poor survival, similar to the bone-fide BC-specific lncRNA, *HOTAIR* [21]. Moreover, for the LNN group we found not only a positive correlation between 8q24 amplification and *CCAT2* expression, but also a significant inverse correlation of *CCAT2* levels with *ESR1* and *PGR* levels. The *ESR1* and *PGR* are essential predictive markers for BC, as *ERS1* positive BCs are known to have a better prognosis and to respond to endocrine therapy. Consequently, this implies that LNN breast cancer patients having elevated levels of *CCTA2* might not benefit from endocrine therapy.

On the other hand, contrary to our expectations we did not observe any correlation with the rs6983267 genotypes for neither of our patient groups and also our *in vitro* assays did not show any differences between the two genotypes. Of note, the SNP rs6983267 has been associated with risk of developing prostate and colon cancer in previous studies [15-17], as well as inflammatory breast cancer (IBC) [19], however, because our study groups were not selected to include a substantial number of IBC patients, this might be interesting to explore. All together, these data and the recently published ones from our group in CRC [12], support the concept that *CCAT2* and rs6983267 are mainly associated with CRC and might be of interest for a specific sub group of BC.

More specifically, *CCAT2* appears to be involved in a pathway explicitly used by non-anthracyclines. In accordance with this hypothesis, our *in vitro* chemosensitivity assays revealed increased resistance to 5'FU treatment for cells overexpressing *CCAT2* with both G and T RNA allele compared to the control cells. Similar results were obtained when examining the migration potential of *CCAT2* overexpressing cells *in vitro*. In both cellular models, MDA-MB-231 and MDA-MB-436, high *CCAT2* levels upregulated cell migration. Taken together, the results suggest *CCAT2* modulates *in vitro* migration and chemoresistance in a SNP-independent fashion. The molecular mechanism driving this regulation is still unclear. We speculate a possible involvement of *MYC* for the observed metastatic phenotype of high *CCAT2* cells, in light of our previous findings in colon cancer [12] and of the documented presence of a large chromatin-loop bringing the SNP rs6983267 in the vicinity of the *MYC* oncogene in two IBC cell lines (SUM149 and SUM190). On the other hand, the increased chemoresistance of the *CCAT2* overexpressing cells may not be mediated by *MYC*, considering recent reports [22, 23], but rather by *TCF4/β-catenin* signaling. Our group has recently exposed the reciprocal regulatory mechanism between *CCAT2* and the Wnt pathway and furthermore, various studies have shown a positive correlation of *TCF4/β-catenin* expression with chemoresistance to 5'FU, mostly in colon cancer, but also in BC [24-26]. Nonetheless, additional functional studies are required for complete understanding of the mechanisms.

In conclusion, our results suggest that *CCAT2* may represent a valuable predictive marker of clinical outcome (shorter MFS and OS) for a specific subgroup of BC patients, for which high levels of this long non-coding RNA will indicate that these patients will not benefit from CMF adjuvant chemotherapy.

## MATERIALS AND METHODS

### Patients

The Erasmus Medical Center (EMC) patient cohort. A protocol for studying biological markers associated with disease outcome was approved by the medical ethics committee of the Erasmus MC Rotterdam, The Netherlands (MEC 02.953). The study, for which written consent was not required, was performed in accordance with the Code of Conduct of the Federation of Medical Scientific Societies in the Netherlands (http://www.federa.org). To avoid bias, tumors were selected from the tumor bank at the Erasmus Medical Center (Rotterdam, The Netherlands) by processing all available frozen tumor samples from female patients with BC who entered the clinic during 1979–2000 from whom detailed clinical follow-up was available. Control normal breast tissue was collected from 15 cancer patients that either underwent prophylactic mastectomy (n=5) or in which the breast tissue was removed at a distance from the primary tumor (n=10). Further inclusion criteria for the BC tissues were as follows: >100 mg frozen tissue available, invasive BC, no previous other cancer (except basal cell skin cancer or cervical cancer stage Ia/Ib), no 2nd primary breast tumor at first relapse, adjuvant treatment for the lymph node positive (LNP) patients, no adjuvant systemic treatment for the lymph node negative (LNN) patients. Of the remaining samples, 8% were excluded because of poor RNA quality and 18% because the genomic DNA contamination was considered too high to guarantee correct evaluation of the non-intron spanning (monoexonic) *CCAT2* gene (see also below).

The remaining 997 patients were treated either with breast-conserving surgery (48%) or with modified mastectomy (52%). Six hundred seventy-five patients (68%) received adjuvant radiotherapy. All 376 LNP patients included in this study were treated with adjuvant systemic therapy, of whom 168 received hormonal therapy, 188 chemotherapy, and 20 combination therapy. Four hundred sixty-six patients (47%) developed a distant metastasis and count as events in the analysis for metastasis-free survival (MFS). Fifty-eight patients died without evidence of distant metastasis and were censored at last follow-up in the analysis of MFS. Three hundred eighty-one patients (38%) died after a previous relapse. Thus, 439 patients (44%) were counted as events in the analysis of overall survival (OS). Tumor staging was according to the Union Internationale Contre le Cancer (UICC) tumor node metastasis classification [27]. Wherever possible, the study has been reported in line with the Reporting Recommendations for Tumor Marker Prognostic Studies guidelines [28]. Other relevant patient and tumor characteristics are listed in Table 1.

The Oncology Institute Cluj-Napoca (OICN) patient and control cohorts. Fifty-six individuals enrolled in the study, with ages between 30 and 67, gave their written consent for sample collection and the molecular analysis and the study was approved by the Institutional Research Ethics Committee. The samples were collected between November 2008 and March 2013. BC diagnosis was confirmed by mammography or ultrasound with tumors over 3 cm diameter. Pathology analysis and staging was done according to American Joint Committee on Cancer (AJCC) criteria. ER, PR and Her2/neu status was analyzed by immunohistochemistry (IHC). Patients with Her2/neu 2+ were tested for gene amplification with chromogenic *in situ* hybridization (CISH) for validation. The patient's cohort included tumors with one positive receptor, except for one case, which was a triple negative breast tumor. All tumor samples were obtained from patients with ductal invasive carcinomas DCI (n=30), two of them being combined: one with mucinous and one with cribriform carcinomas. After initial diagnosis, 25 patients had neoadjuvant chemotherapy, while 5 patients underwent sectorectomy or mastectomy immediately after initial diagnosis without neoadjuvant chemotherapy. Of these 25 patients, 13 patients followed neoadjuvant chemotherapy with epirubicin and cyclophosphamide (EC), 7 patients had cyclophosphamide and doxorubicin (AC) and 5 patients EC/AC (epirubicin/doxorubicin + cyclophosphamide) + (taxotere) TXT. As by August 2013 all patients were alive. Normal breast tissues as control samples (n=26) were collected from patients diagnosed with fibrosis (n=5), fibro adenomas (n=4) and one phyllodes tumor, all collected by core biopsies or open excisional biopsy, or with invasive carcinomas (n=16) collected by surgical procedures. All samples were snap frozen in liquid nitrogen immediately after collection and stored until RNA extraction.

### Tissue processing

The EMC patient cohort. The tissue processing and the estimation of invasive tumor cells was performed as previously described [29, 30]. Only specimens with at least 30% of the nuclei of epithelial tumor cell origin and distributed uniformly over at least 70% of the hematoxylin-eosin–stained tissue section area were included.

The OICN patient and control cohorts. Freshly harvested BC and non-cancer tissues were snap frozen in liquid nitrogen and disrupted using a mortar and pestle, until a fine powder was obtained.

### RNA isolation, cDNA synthesis and RT-qPCR assay

The EMC patient cohort. RNA isolation, cDNA synthesis, quantification of specific (m)RNA species, and quality control checks were done as described in detail [30]. Real-time RT-PCR (RT-qPCR) was performed in an ABI Prism 7700 Sequence Detection System (Applied Biosystems) and a Mx3000P™ Real-Time PCR System (Stratagene). PCR reactions were done in a final volume of 25 μl containing cDNA synthesized from 5 to 15 ng of total RNA, 330 nM forward and reverse primer and 12.5 μl Absolute™ QPCR SYBR® Green (Abgene Limited, Epsom, UK). After 15 minutes of denaturation and activation of the Taq-DNA polymerase, PCR products were amplified in 35 cycles with 15 seconds of denaturing at 95°C, 30 seconds of annealing at 62°C followed by data acquisition at 62°C. To correct for possible contribution of traces of genomic DNA present in the total RNA samples, we measured the levels of an unrelated intronic sequence, *C17* on chromosome 17q25 at the same PCR conditions and subtracted from the *CCAT2* transcript levels. Specificity of the *CCAT2* RNA transcript levels after correction for genomic DNA contribution with our quantitative *C17* genomic DNA PCR assay was further validated by RT-qPCR in a set of breast tumor samples before and after DNAse I treatment and by comparing levels measured in cDNA generated in the absence and presence of reverse transcriptase. When amplification rounds for *CCAT2* exceeded 35 cycles, which was the case for 24% of the remaining samples, quantities were considered to be undetectable and were set at 50% of the expression level measurable at the detection threshold (0.00001). Primer sequences for *ESR1*, *PGR*, and the reference genes have all been described, as have the PCR reactions and validations performed to ensure PCR specificity [30]. To measure concentrations of the proliferation marker *Ki-67*, we used the Hs00606991_m1 Assay-on-Demand from Applied Biosystems. For *MYC* we used the Hs00905030_m1 Assay-on-Demand. Concentrations of the target genes, expressed relative to our reference gene set [low-abundance reference gene hydroxymethylbilane synthase (*HMBS*, formerly porphobilinogen deaminase, *PBGD*), medium-abundance hypoxanthine phosphoribosyltransferase (*HPRT1*), and high-abundance β2-microglobulin (*B2M*)], were quantified as follows: mRNA target = 2^Ct reference gene set – Ct target gene^, as described [30]. All primer sequences are available in Supplementary Table 1.

The OICN patient and control cohorts. The samples were lysed using TriReagent and homogenized with a Rotor-stator homogenizer. RNA extraction was further carried according to classical phenol-chloroform extraction protocol. The total RNA was quantified with NanoDrop ND-1000 for quantity and Lab-on–Chip Bioanalizer for quality. Only samples with RIN greater than 7.5 were considered for further experiments. 1 μg of total RNA were mixed with 2 μl of DNase buffer, 1 μl of Turbo DNAse (Ambion), 0.5 μl of RNAse Inhibitor (Roche) and RNase free H_2_O to a final volume of 20 μl and incubated for 30 min at 37°C. The DNAse was inactivated for 5 min with 2 μl of DNAse Inactivation Reagent, samples were centrifuged and the RNA was transferred to fresh tubes. Before proceeding with the cDNA synthesis, RNA integrity after DNAse treatment was confirmed as described above.

Eight μl of DNAse treated RNA was used for cDNA synthesis using Transcriptor FirstStrand cDNA synthesis kit (Roche) according to the manufacturer instructions. RNA was diluted to a volume of 11 μl and incubated with 2 μl of Random hexamer primers at 65°C for 10 min to remove secondary structures. The cDNA synthesis mix consisted of 4 μl of buffer, 2 μl of dNTPs, 0.5 μl of RNAse Inhibitor and 0.5 μl of reverse transcriptase. The reverse transcription reaction was performed in a heated lid thermocycler for 10 min at 25°C, followed by 30 min at 55°C. The reverse transcriptase was inactivated by heating the samples at 85°C for 5 min. Samples with the RNA only were treated in the same manner and used as negative controls. Real time RT-PCR was performed in a LightCycler 480 apparatus using LightCycler 480 DNA SYBR Green I Master (Roche) with a primer concentration of 0.4 μM in a 10 μl reaction as instructed by the manufacturer and 20 ng of cDNA were added to the mastermix. The C_t_ values were assessed using the automated second derivative max analysis. For the samples that exceeded 35 cycles of amplification, *CCAT2* was considered not to be expressed. The primer sequences for *CCAT2* were identical to the ones used for the EMC patient cohort. *U6* and *HPRT1* were used as reference genes and the primer sequences are found in Supplementary Table 1. Results were analyzed using the 2^-deltaCt^ method.

### Copy number alterations

Genomic DNA from 313 primary breast tumors LNN BC patients, from which 226 are included in this study to correlate copy number with *CCAT2* mRNA expression, was hybridized to Affymetrix GeneChip@ Human 100K SNP Arrays as described before [31]. The median of the mean copy numbers computed from each SNP's interquartile copy number estimates of the 313 breast tumors was 2.1, consistent with the general assumption that the majority of the genome is diploid. The DNA copy numbers for 12 SNP loci covering chr8:128,443,462-128,487,117 in the human genome were analyzed to identify samples whose copy number alterations (CNAs) were informative for gain, which was set at 1 unit gain over the diploid copy number of 2.1, in this 8q24 region.

### Tissue microarray (TMA)

Tissue specimens were obtained at the University of Texas, MD Anderson Cancer Center (MDACC) from women prospectively enrolled into LAB 08-0700, a blood and tissue based study examining biomarkers of breast cancer risk. This study enrolls women with a breast cancer diagnosis OR women with mammographic abnormality undergoing stereotactic biopsy. Paraffin embedded tissue blocks from either the surgical specimen (cancer patients) or stereotactic biopsy (non-cancer controls) were selected for the creation of tissue microarrays. For each case, up to 5, 1 mm cores were transferred to a TMA block. After processing, unstained slides from the TMA block were used for the *in situ* hybridization as detailed below. Approval for this study was obtained from the institutional review board of MDACC.

### *In situ* hybridization (ISH)

TMA slides were incubated with either a double-DIG-labeled *CCAT2* probe or control *U6* snRNA probe (Exiqon) and detected as previously described [12].

### Cell culture

Specifics of the breast cancer cell line panel used at EMC to evaluate the expression of *CCAT2* RNA have been described [32]. Human BC MDA-MB-231 and MDA-MB-436 cell lines used for the *in vitro* manipulation experiments were obtained from the American Type Culture Collection and grown as suggested by the supplier. Cells were cultured at 37^0^ C in 5% CO_2_. All cell lines were validated using STR DNA fingerprinting.

### Virus production

The stable MDA-MB-231 cells for overexpressing *CCAT2* were prepared as previously described [12]. Briefly, the *CCAT2*-containing genomic region was amplified with genomic DNA with *Pfu* polymerase (Invitrogen) and cloned it into the pMX vector (Cell Biolabs). The *CCAT2*-containing retrovirus was then produced in 293 GP2 cell lines and the virus-containing supernatant was used to infect MDA-MB-231 cells. After infection, MDA-MB-231 cells were grown in complete media containing puromycin (1 μg/ml).

### Plasmid production and transient reverse transfection

The same sequences as used for the pMX retrovirus were cloned into a pcDNA 3.1 vector (Invitrogen) [12] and these vectors were further used for transient reverse transfection. Briefly, the transfection mix was prepared using Lipofectamine2000, according to the manufacturer's protocol for a final concentration of 50 nM plasmid/well. During the incubation period for forming the transfection complex, the cells were prepared at 80-90% confluence and added to the transfection mix at the end of the incubation period. After 24 hours cells were harvested, a part was further used for migration assay and the remainder was used for assessment of transfection efficiency by RT-qPCR.

### *In vitro* migration assay

Migration assay was performed as previously described [12]. Briefly, 100 μl of serum-free media containing the cells (60 000 cells/insert for MDA-MB-436 and 55 000 cells/insert for MDA-MB-231) were seeded onto the top of gelatin-coated insert and 500 μl of media with serum was added to the bottom well. Cells were left to migrate 8 hours for MDA-MB-231 and 20 hours for MDA-MB-436, the optimum migration conditions for these cell lines, respectively. The cells that migrated to the bottom of the well were fixed, stained and counted using a microscope. For each well, 6 different fields were counted and the average number of cells was determined. The experiments were performed in triplicates.

### MTT (3-[4,5-dimethylthiazol-2-yl]-2,5-diphenyltetrazolium bromide) assay

*In vitro* chemoresistance to 5'Fluorouracil (5'FU) of MDA-MB-231 *CCAT2* clones was assessed by MTT. Briefly, cells were plated 24 hours prior to treatment in 96 well microculture plates. After cells were adherent, 3 different doses of the drug were added to the supernatant without medium change. After 120 hours, the MTT reagent (Sigma) was added to each well and incubated for 3 hours at 37° C. The optical density (OD) was read at 570 nm on a microplate spectrophotometer and growth values (%) were calculated as followed (OD_treated cells_/OD_untreated cells_) × 100. The experiments were performed in quadruplicate.

### Statistics

All the results derived from the *in vitro* experiments were expressed as the mean ± SD for at least two separate experiments in triplicate or quadruplicate. For correlations with *in vitro* findings, data analysis was performed with SPPS and GraphPad Prism software. For correlations with clinical data, the STATA statistical package, release 12 (STATA Corp.) and SPSS 20.0 were used. The Shapiro-Wilk test was applied to verify if *CCAT2* expression follows a normal distribution. Accordingly, t-test, respectively ANOVA test (depending on the number of groups considered) or the nonparametric test Mann-Whitney-Wilcoxon, respectively Kruskal-Wallis was applied to assess the relationship between *CCAT2* expression and other characteristics. The strengths of the associations between continuous variables were tested with the Spearman rank correlation (*r*_s_). Variables were either log-transformed or Box-Cox–transformed to reduce the skewness. Because even after these transformations *CCAT2* RNA levels were not normally distributed in the n=997 clinical BC sample set (Fig 1d), clinical evaluations were performed after dividing *CCAT* RNA levels into 4 equally sized groups, thus also taking into account the 24% samples with undetectable levels of *CCAT2*. The prognostic values of the clinical and biological variables were tested with MFS and OS as the endpoint in the univariate, multivariate, and interaction analyses, with the use of the Cox proportional hazards model. The hazard ratio (HR) and its 95% confidence interval (CI) were derived from these results. We used Kaplan-Meier survival plots and log-rank tests for trend to assess the differences in time of the predicted high and low risk groups of patients. All tests were 2-sided, and *P*<0.05 was considered statistically significant.

## Supplemental Figures and Tables


