# Supplementary material for: CCAT2, a novel long non-coding RNA in breast cancer: expression study and clinical correlations
Source: Oncotarget. 2013 Sep 18;4(10):1748–62. doi: 10.18632/oncotarget.1292 (PMC3858561; doi:10.18632/oncotarget.1292)

**Supplementary figures and tables:**

***CCAT2*, a novel long non-coding RNA in breast cancer: expression study and clinical correlations**

Roxana S Redis^*^, Anieta M Sieuwerts^*^, Maxime P Look, Oana Tudoran, Cristina Ivan,

Riccardo Spizzo, Xinna Zhang, Vanja de Weerd, Masayoshi Shimizu, Hui Ling, Rares Buiga,

Victor Pop, Alexandru Irimie, Riccardo Fodde, Isabella Bedrosian, John WM Martens, John A Foekens, Ioana Berindan-Neagoe^#^, George A Calin^#^

**CONTENTS**

**Supplementary Table 1.** Primer sequences

**Supplementary Table 2**. Associations of *CCAT2* RNA levels with clinical, biological and histo-morphological factors.

**Supplementary Figure 1**. Northern Blot image of human colon and BC cell lines probed for *CCAT2*.

**Supplementary Figure 2.** ISH images of *CCAT2* in normal breast and BC tissue.

**Supplementary Figure 3.** Relationship between *CCAT2* RNA and MFS.

**Supplementary Table 1.** Primer sequences

| Gene/SNP | Study cohort | Description | F Primer sequence | | R Primer sequence | | Ref seq | Product size (bp) | |
| --- | --- | --- | --- | --- | --- | --- | --- | --- | --- |
| *CCAT2* | EMC  OICN | 8q24 non coding long RNA | CCCTGGTCAAATTGCTTAACCT | | TTATTCGTCCCTCTGTTTTATGGAT | | NT_008046.16 | 66 | |
| *C17* | EMC | to correct for gDNA traces | CTCTGGGAACAACTCTGGGATGAGG | | ACTCAGGTGGTCCCAGGAAGTGTGG | | NT_010783.15 | 136 | |
| *ESR1* | EMC | Estrogen receptor | ATCCTACCAGACCCTTCAGTG | | GCCAGACGAGACCAATCATC | | XM_045967 | 186 | |
| *PGR* | EMC | Progesterone receptor | CAAGTTAGCCAAGAAGAGTTC | | ACTTCGTAGCCCTTCCAAAG | | NM_000926 | 78 | |
| *ERBB2* | EMC | HER2/neu | GTCTACAAGGGCATCTGGAT | | GTGGATGTCAGGCAGATGC | | NM_004448 | 179 | |
| *HPRT1* | EMC | reference gene | TATTGTAATGACCAGTCAACAG | | GGTCCTTTTCACCAGCAAG | | NM_000194 | 192 | |
| *HMBS* | EMC | reference gene | CATGTCTGGTAACGGCAATG | | GTACGAGGCTTTCAATGTTG | | NM_000190 | 139 | |
| *B2M* | EMC | reference gene | CTTTGTCACAGCCCAAGATAG | | CAATCCAAATGCGGCATCTTC | | NM_004048 | 83 | |
| *HPRT1* | OICN | reference gene | TGACACTGGCAAAACAATGCA | | GGTCCTTTTCACCAGCAAGCT | | NM_000190 | 94 | |
| *U6* | OICN | reference gene | CTCGCTTCGGCAGCACA | | AACGCTTCACGAATTTGCGT | | NM_000194 | 94 | |
|  |  | **Taqman assay** |  | |  | |  |  | |
| *MKI67* | EMC | Hs00606991_m1 | |  | |  | NM_002417 | | 137 |
| *MYC* | EMC | Hs00905030_m1 | |  | |  | NM_002467 | | 87 |
| *rs6983267* | EMC | C_29086771_20 | GTCCTTTGAGCTCAGCAGATGAAAG[**G/T**]CACTGAGAAAAGTACAAAGAATTTT | | | | Chr.8:128413305 on NCBI Build 37 | | |
| *rs13281615* | EMC | C_1332250_10 | GTAACTATGAATCTCATCAAAAGAA[**A/G**]GCAGAACGCAGATATTCTGAGTAGG | | | | Chr.8:128355618 on NCBI Build 37 | | |

**Supplementary Table 2**. Associations of *CCAT2* RNA levels with clinical, biological and histo-morphological factors.

|  |  |  | **All patients** | | | |  | **All lymph node positive patients** | | | |  | **All LNP patients that received adjuvant CMF** | | | |
| --- | --- | --- | --- | --- | --- | --- | --- | --- | --- | --- | --- | --- | --- | --- | --- | --- |
|  | | No of | | *%* |  | | No of | | % |  | | No of | | % |  | |
| **Characteristic** | | patients | |  | ***CCAT2* RNA** (x10^-2^**)** | | patients | |  | ***CCAT2* RNA** (x10^-2^**)** | | patients | |  | ***CCAT2* RNA** (x10^-2^**)** | |
|  | |  | |  | median | inter-quartile |  | |  | median | inter-quartile |  | |  | median | inter-quartile |
| **All patients in this cohort** | |  | 997 | *100%* | 0.61 | 2.98 |  | 376 | *100%* | 0.43 | 1.17 |  | 134 | *100%* | 0.41 | 1.26 |
| **Age (years)** | |  |  | |  |  |  |  |  |  |  |  |  |  |  |  |
|  | ≤40 |  | 140 | *14%* | 0.69 | 5.12 |  | 56 | *15%* | 0.57 | 2.28 |  | 35 | 26% | 0.58 | 2.11 |
|  | 41-55 |  | 394 | *40%* | 0.57 | 3.07 |  | 173 | *46%* | 0.40 | 1.19 |  | 96 | 72% | 0.37 | 0.98 |
|  | 56-70 |  | 313 | *31%* | 0.56 | 2.36 |  | 112 | *30%* | 0.38 | 1.03 |  | 3 | 2% | 1.11 | 3.27 |
|  | >70 |  | 150 | *15%* | 0.77 | 2.82 |  | 35 | *9%* | 0.29 | 1.07 |  | 0 | 0% |  |  |
|  |  |  |  |  | *P* =0.19^‡^ |  |  |  |  | *P* =023^‡^ |  |  |  |  | *P* =0.56^‡^ |  |
| **Menopausal status** | |  |  |  |  |  |  |  |  |  |  |  |  |  |  |  |
|  | premenopausal |  | 460 | *46%* | 0.61 | 3.69 |  | 193 | *51%* | 0.49 | 1.38 |  | 119 | *89%* | 0.45 | 1.46 |
|  | postmenopausal |  | 537 | *54%* | 0.59 | 2.40 |  | 183 | *49%* | 0.31 | 0.96 |  | 15 | *11%* | 0.37 | 0.79 |
|  |  |  |  |  | *P* =0.06^§^ |  |  |  |  | ***P* =0.05^§^** |  |  |  |  | *P* =0.38^§^ |  |
| **ER mRNA status**^≠^ | |  |  |  |  |  |  |  |  |  |  |  |  |  |  |  |
|  | negative, < 0.2 |  | 230 | *23%* | 1.19 | 4.78 |  | 74 | *20%* | 0.43 | 1.50 |  | 25 | *19%* | 0.67 | 1.47 |
|  | positive, ≥ 0.2 |  | 767 | *77%* | 0.54 | 2.16 |  | 302 | *80%* | 0.43 | 1.06 |  | 109 | *81%* | 0.40 | 1.07 |
|  |  |  |  |  | ***P <*0.001^‡^** |  |  |  |  | ***P =0.01*^‡^** |  |  |  |  | *P*=0.62^‡^ |  |
| **PR mRNA status**^≠^ | |  |  |  |  |  |  |  |  |  |  |  |  |  |  |  |
|  | negative, < 0.1 |  | 396 | *40%* | 0.83 | 4.09 |  | 151 | *40%* | 0.53 | 1.32 |  | 45 | *34%* | 0.53 | 1.46 |
|  | positive, ≥ 0.1 |  | 601 | *60%* | 0.49 | 2.24 |  | 225 | *60%* | 0.35 | 1.04 |  | 89 | *66%* | 0.40 | 1.16 |
|  |  |  |  |  | ***P <*0.001^‡^** |  |  |  |  | *P*=0.11^‡^ |  |  |  |  | *P*=0.98^‡^ |  |
| **Grade** | |  |  |  |  |  |  |  |  |  |  |  |  |  |  |  |
|  | poor |  | 531 | *53%* | 0.65 | 3.44 |  | 223 | *59%* | 0.50 | 1.24 |  | 70 | *52%* | 0.54 | 1.98 |
|  | unknown |  | 282 | *28%* | 0.56 | 2.41 |  | 99 | *26%* | 0.29 | 0.86 |  | 42 | *31%* | 0.24 | 0.79 |
|  | Moderate/good |  | 184 | *18%* | 0.57 | 2.44 |  | 54 | *14%* | 0.40 | 1.15 |  | 22 | *16%* | 0.28 | 0.58 |
|  |  |  |  |  | *P* =0.38^║^ |  |  |  |  | *P* =0.41^║^ |  |  |  |  | *P* =0.08^║^ |  |
| **Tumor size** | |  |  |  |  |  |  |  |  |  |  |  |  |  |  |  |
|  | pT1, ≤2 cm |  | 377 | *38%* | 0.57 | 2.20 |  | 86 | *23%* | 0.31 | 1.04 |  | 36 | *27%* | 0.30 | 0.72 |
|  | pT2, >2-5 cm + unknown | | 534 | *54%* | 0.64 | 3.46 |  | 227 | *60%* | 0.45 | 1.23 |  | 80 | *60%* | 0.45 | 1.56 |
|  | pT3, >5 cm + pT4 |  | 86 | *9%* | 0.52 | 2.59 |  | 63 | *17%* | 0.48 | 1.23 |  | 18 | *13%* | 0.51 | 1.62 |
|  |  |  |  |  | *P* =0.24^║^ |  |  |  |  | *P* =0.37^║^ |  |  |  |  | *P* =0.83^║^ |  |
| **Lymph nodes involved** | |  |  |  |  |  |  |  |  |  |  |  |  |  |  |  |
|  | no, (LNN) |  | 621 | *62%* | 0.84 | 4.60 |  | 0 | *0%* |  |  |  | 0 | *0%* |  |  |
|  | yes, (LNP), 1 to 3 |  | 189 | *19%* | 0.40 | 1.24 |  | 189 | *50%* | 0.40 | 1.24 |  | 92 | *69%* | 0.40 | 1.39 |
|  | yes, (LNP), >3 |  | 187 | *19%* | 0.45 | 1.11 |  | 187 | *50%* | 0.45 | 1.11 |  | 42 | *31%* | 0.51 | 1.16 |
|  |  |  |  |  | ***P* <0.001^║^** |  |  |  |  | *P*=0.98^§^ |  |  |  |  | *P*=0.14^§^ |  |
| **Histological type**^†^ | |  |  |  |  |  |  |  |  |  |  |  |  |  |  |  |
|  | IDC |  | 533 | *53%* | 0.56 | 2.39 |  | 197 | *52%* | 0.40 | 1.23 |  | 23 | *17%* | 0.37 | 1.57 |
|  | DCIS + IDC |  | 151 | *15%* | 0.50 | 1.97 |  | 68 | *18%* | 0.39 | 0.83 |  | 67 | *50%* | 0.46 | 0.87 |
|  | ILC |  | 85 | *9%* | 0.61 | 3.58 |  | 31 | *8%* | 0.96 | 2.47 |  | 9 | *7%* | 0.52 | 3.49 |
|  | mucinous |  | 29 | *3%* | 0.47 | 1.27 |  | 8 | *2%* | 0.09 | 0.27 |  | 6 | *4%* | 0.05 | 0.10 |
|  |  |  | |  | *P* =0.50^║^ |  |  | |  | ***P* =0.01^║^** |  |  | |  | *P* =0.11^║^ |  |

≠ ER+ and/or PR+ with real-time PCR cut point used for ER, 0.2 and PgR, 0.1 (mRNA levels relative to reference gene set).

† Only data for the 4 most common histological subtypes are presented in this table IDC; infiltrating ductal carcinoma, DCIS; ductal carcinoma in situ, ILC; infiltrating lobular carcinoma.

* Due to missing data numbers do not add up to 997.

‡ *P* for Spearman rank correlation test.

§ *P* for Mann-Whitney U test.

║ *P* for Kruskal-Wallis test, including a Wilcoxon-type test for trend when appropriate.

**Supplementary Figure 1**. Northern Blot image of human colon and BC cell lines probed for *CCAT2*. Migration of the marker is shown on the right.


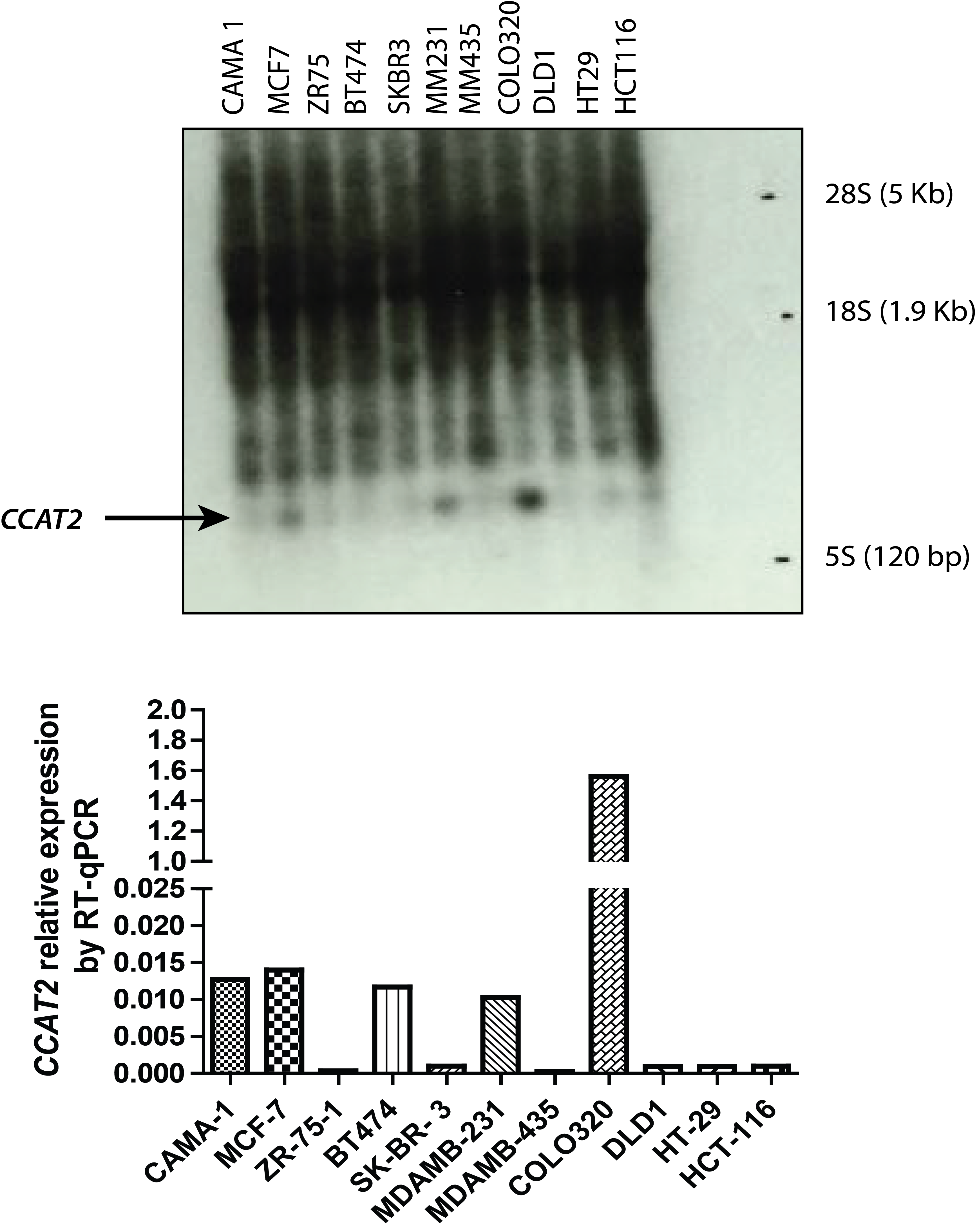


**Supplementary Figure 2.** a) Breast carcinoma with strong expression of *CCAT2* in epithelial (cancer) cells. Epithelial islands (black arrows) are surrounded by rare inflammatory cells (intense blue dots). b). Breast carcinoma *in situ* (not invasive) located in a dilated duct (gray arrows); please note the intense staining comparable to the invasive carcinoma depicted in a). c). Normal breast tissue with apocrine metaplasia (black star); note the stronger and heterogeneous nuclear staining compared to the cytoplasm. d). Normal breast tissue represented by a large duct (galactiferous) (gray stars); nuclei are more intensively stained than cytoplasm. Rare stromal cells and 2-3 inflammatory cells surround the duct.


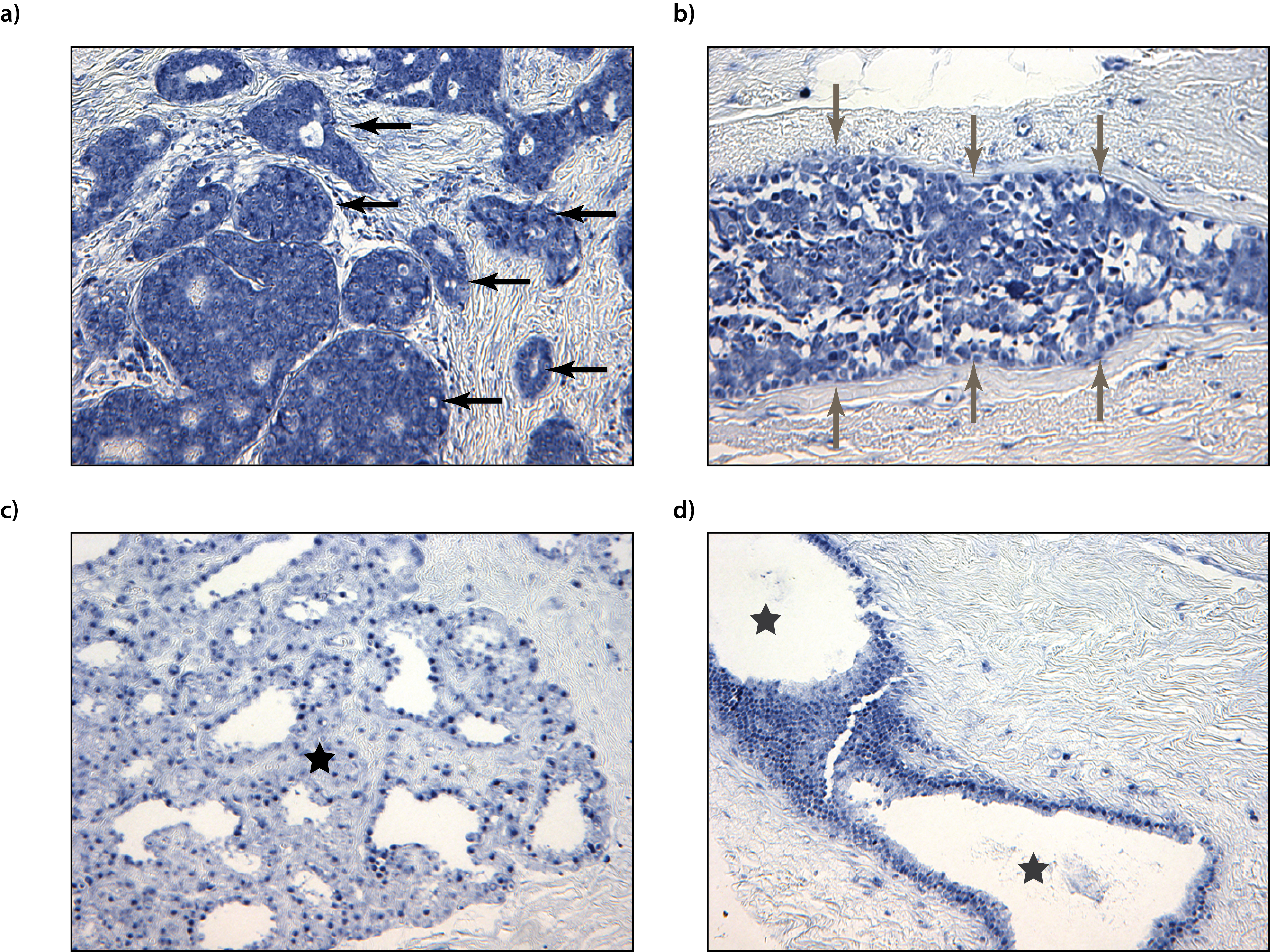


**Supplementary Figure 3.** Relationship between *CCAT2* RNA and MFS. RNA levels divided into 4 parts (negative to low - green, intermediate - blue and high – red) for the 134 LNP primary BC patients that received systemic adjuvant non-anthracycline (CMF). Patients at risk are indicated, as well as the log Rank *P*-value.


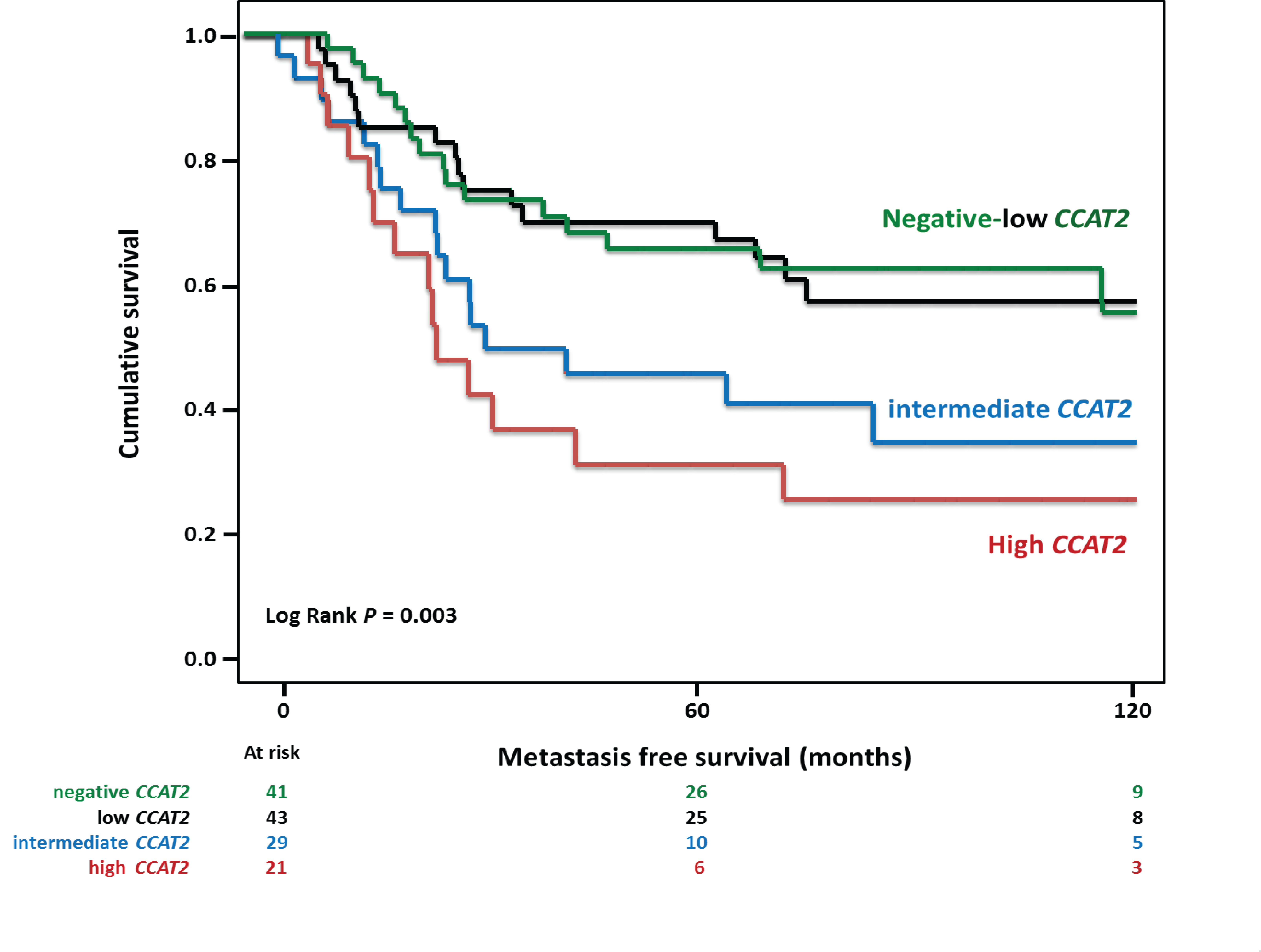

Supplement: Supplementary file 1 [file oncotarget-04-1748-s001.docx]
